# Supplementary material for: Shortage and unequal distribution of infectious disease specialists in Japan: How can we refine the current situation?
Source: PLoS One. 2023 Oct 20;18(10):e0291677. doi: 10.1371/journal.pone.0291677 (PMC10588892; doi:10.1371/journal.pone.0291677)
Supplement: Attachment — Submitted filename: revise letter.docx [file pone.0291677.s001.docx]

25th July 2023

Dear Dr. Takashi Watari

Academic Editor

PLOS ONE

**Manuscript Number: PONE-D-23-04516-R1**

**Biased allocation of infectious diseases specialists in Japan**

We are grateful to both the Editor and Referees for their constructive critique and recommendations to improve our manuscript. We have made every effort to address the issues raised and have responded to all comments. The manuscript has been rechecked and the necessary changes have been made in accordance with the referees’ suggestions. The changes to the revised manuscript have been indicated with red font. I look forward to working with you and the referees to move this manuscript closer to publication. Please find our point-by-point responses to the Editor’s and Referee’s comments below.

**Note:**

**#1 The title has been changed to address more clearly what the author state in this manuscript.**

**#2 Author’s affiliation has been changed because it was changed during the submission process.**

**#3 Overall, English grammar have been checked again and revised as appropriate partially by the support of ChatGPT.**

Thank you for your consideration. I look forward to hearing from you.

Sincerely yours,

Hideharu Hagiya, M.D., Ph.D., CTH, CIC

Department of Infectious Diseases, Okayama University Hospital

2-5-1 Shikata-cho, Kita-ku, Okayama 700-8558, Japan

Tel: +81-86-235-7342

Fax: +81-86-235-7345

E-mail: hagiya@okayama-u.ac.jp

**Reviewer #1: General comments**
This article discusses the uneven distribution of infectious disease physicians in Japan. This is an issue that has not been discussed much until now, but with the COVID-19 pandemic, it has become an issue that should be seriously considered. This is an important paper that provides important suggestions for medical policy.
However, it is also true that many issues remain that need to be corrected, and these should be addressed through the following comments.

**Major comments**
1. The author's opinion is stated in the Introduction, and in some places the discussion is based on facts that are not with appropriate evidence. This point needs to be addressed.

**Comment #1**

According to your suggestions, the manuscript was entirely revised to avoid stating the authors’ opinions in the Introduction.

2. I agree that it is important to increase the number of female infectious disease physicians in the future, as it is stated that there are few women among infectious disease physicians. However, there is a problem with the reason that housework and being an infectious disease physician can be compatible. This is because I think it could be misinterpreted that the author thinks it is common for women to do household tasks.

**Comment #2**

Thank you for your opinion. I am NOT a male chauvinist who insists the household tasks should be allocated to women. However, in Japanese society and family, it is still a general thought that domestic chores be done by females. Although men are doing housework these days, an imbalance in the burden of housework between men and women does exist. It is not a question of whether it is right or wrong; just it is a fact. And, this fact would inevitably affect female doctors when choosing their future careers.

3. It is stated that the gender and age of the infectious disease physicians were obtained from the Japanese Association for Infectious Diseases. If so, I think that the authors need to provide the reason why they were able to receive that data from the Japanese Society of Infectious Disease. The authors have stated that they have not undergone ethical review in conducting their research, but I think there is a possibility that they handled personal information. If the authors received post-processed data from the Japanese Society for Infectious Diseases, there would be no issue.

**Comment #3**

Thank you for addressing the ethical concern. In response to my request, anonymized data was shared by the Japanese Society for Infectious Diseases.

4. The small number of infectious disease physicians in Japan has been raised as a problem, and information on how the situation is in other developed countries is considered important. Europe, the U.S., and Australia seem to have more infectious disease physicians per 100,000 people than Japan, while France, for example, does not. Please incorporate the perspective of how it is in other countries.

**Comment #4**

According to your advice, the following sentences were added in the second paragraph of Discussion section; “As of November 2022, prevalence of ID specialists in Japan was 1.35 per 100,000 population, which was approximately three-times higher than in Korea (0.47 in 2019) [8]. Although clear data for other developed countries is unavailable, the situation appears to vary in each country. For example, the results of the 2023 ID fellowship match in the United Staes were disastrous, with 44% of ID fellowship programs remaining unfilled [9], indicating a potential future decline in the number of ID specialists. While, there has been an increasing interest in ID specialization among medical students in France recently [10], which may contribute to a rise in the number of ID specialists.”

**Minor comments**
Line 59 I think it would be better to state somewhere what is included in Class I infectious diseases.

**Comment**

Thank you for your advice. I have added the following sentence: “Of the various designated diseases, the Class I Infectious Diseases include fatal diseases such as Ebola haemorrhagic fever, Crimean-Congo haemorrhagic fever, Smallpox, South American haemorrhagic fever, Plague, Marburg virus disease, and Lassa fever.”

Line 65 The government requires that "physicians who have been engaged in the practice of infectious diseases" be engaged in Class I DMIs. The problem is that the phrase "engaged in infectious disease practice" is sometimes interpreted expansively and is deceptively used by physicians who have no training in infectious disease practice. Please revise for clarity on this point.

**Comment**

The point addressed is important.

I have added the following sentence: “The government may address that physicians who have been engaged in the clinical practice of infectious diseases should manage such DMIs; however, that is not the case in reality.”

Line 66 It is unusual for the author's opinion to be expressed in the Introduction section. However, the author's concern is valid, so please revise the wording.

**Comment**

Thank you for your advice. I have revised it as follows; “In such a situation, this legal framework might not be the most practical for managing disease outbreaks.”

Line 94 When writing a paper using the EZR software, there are references that should be cited. Please confirm.

**Comment**

Added the relevant citation.

Line 118 In the discussion of the gender ratio of infectious disease specialists, there is a category of "overseas," which I think should be cut.

**Comment**

The comment on “oversea” was deleted as recommended.

Line 164 Is there any evidence that the situation will improve if the government controls the number of infectious disease specialists? Please add this taking into account the outline of the new specialist system.

**Comment**

Thank you for your interest.

Actually, as far as concerned, there is no valid evidence for the usefulness of a government-controlled ID specialist system. In this section, I have raised some possibilities to have full-time ID specialists at DMIs from new perspectives or approaches. The phrases and sentences in the paragraph were fully revised.

Line 171 I think it is unlikely that the fact that female physicians reportedly outperformed male physicians in skill is important to the discussion of this paper.

**Comment**

In that paragraph, I intended to mention our expectation for female physicians in this ID field. The first sentence of the paragraph was revised as follows; “To address these urgent issues, we anticipate active participation of female physicians in the field of infectious diseases.”

Line 175 It is true that the percentage of female among infectious disease specialists is less than that of all physicians, but it is more in dermatology and obstetrics and gynecology. I think it is unclear whether the low percentage of female among infectious disease physicians is really a result of gender inequality.

**Comment**

I appreciate your point. As questioned, it is not clear that unbalance of ID physicians between male and female doctors is merely based on gender inequality. But, herein, the term “gender inequality” was used for the examples of preceding sentences. I have not yet to have a clear conclusion regarding the gender difference, which I am going to tackle. At least, I presently hope as follows: “It is hoped that increased and active participation of female physicians in clinical infectious diseases will help alleviate the problem of insufficient ID specialists.”

Line 177 The number of female authors in medical journals in Japan does not necessarily seem to be decreasing based on a article in 2021 ( J Med Internet Res. 2021 Apr 12;23(4):e25379. Please revise the content to take into account the latest data.

**Comment**

Thank you for sharing the relevant article. I have added the following sentence; “This trend was, however, necessarily not true in Japan, where publications on COVID-19 by female researchers reportedly increased by 23.7% amid the pandemic”

Line 183 Not all infectious disease physicians are consultants. Infectious disease physicians in large medical offices that have produced many infectious disease physicians work both night shifts and as attending physicians. Furthermore, some infectious disease specialists are affiliated with departments other than the Department of Infectious Diseases. Please revise the wording.

**Comment**

I appreciate your advice. The sentences were revised as follows; “A certain portion of ID specialists work as consultants and do not engage in the duties of the physician in charge. In addition, some of them have no night duties or emergency calls; thus, it is comparatively possible to balance childcare and work as full-time doctors.”

Line 189 It is not clear what role the large number of men as representatives and councilors of infectious disease societies plays in drawing conclusions from the results obtained in this study.

**Comment**

Here, I meant that the majority of leadership positions in ID medical societies were occupied by male doctors. This paragraph is structured following the preceding paragraph, stressing that female ID specialists should be placed in such positions to incorporate womens’ perspectives into the establishment of creating a comfortable working environment for female physicians.

*Thank you for your review.*
